# Linked routine health data enables estimation of transition point to adult healthcare and better assessment of changes in emergency care.

**DOI:** 10.23889/ijpds.v7i3.1798

**Published:** 2022-08-25

**Authors:** Stuart Jarvis, Gerry Richardson, Kate Flemming, Lorna Fraser

**Affiliations:** 1 University of York

## Objectives

To estimate point of transition from paediatric to adult healthcare from routinely collected healthcare records and to use this to compare emergency care use pre- and post transition for young people with life-limiting conditions.

## Approach

Routinely collected healthcare records were obtained from the Clinical Practice Research Datalink. These included linked primary care and hospital (inpatient, outpatient and A&E) records and death and deprivation data. The data were used to identify young people (12-23 years)with life-limiting conditions, diabetes and no long term conditions. Methods were developed to estimate point of transition from paediatric to adult care by classifying treatment specialties recorded in inpatient and outpatient care as paediatric or adult. These were compared and a favoured method selected. Emergency hospital care use was then compared, pre- and post-transition to adult care for the three groups.

## Results

The last inpatient or outpatient record classified as paediatric care was chosen as the transition point. Simulation showed that this had the potential for far greater sensitivity to changes at transition (~20% difference in detected effect size) than using a simple age cut-off. Application of the method to the data showed significant increases in emergency inpatient admissions (by 29%, 95% confidence interval 14-46%) and A&E visits (by 24%, 95% confidence interval 12-38%) post- compared to pre-transition in the life-limiting conditions group, but no increases for the diabetes or no long term conditions groups, suggesting that transition has little effect for these groups.

## Conclusion

Linked, routinely collected healthcare records, combined with estimating transition point from the data, provide a more sensitive method for detecting changes at transition with reduced risk of misclassification bias. There is an increase in emergency care after transition, with negative effects on young people, families and cost implications for providers.

